# UBE3C Promotes Growth and Metastasis of Renal Cell Carcinoma via Activating Wnt/β-Catenin Pathway

**DOI:** 10.1371/journal.pone.0115622

**Published:** 2015-02-06

**Authors:** Ji Ling Wen, Xiao Fei Wen, Rong Bing Li, Yong Chao Jin, Xue Lei Wang, Lan Zhou, Hui Xing Chen

**Affiliations:** Department of Urology, East Hospital, Tongji University School of Medicine, Shang Hai 200120, China; Van Andel Institute, UNITED STATES

## Abstract

Renal cell carcinoma (RCC) is the most common primary malignancy of the kidney and one of the most lethal genitourinary malignancies. Clear-cell renal cell carcinoma (ccRCC) has an extremely poor prognosis because of a high potential for tumor growth, vascular invasion, metastasis and recurrence. Unfortunately, the mechanism of RCC growth and metastasis is not well understood. In this report, we for the first time demonstrated ubiquitin protein ligase E3C (UBE3C) as a driving factor for RCC growth and metastasis. UBE3C expression was increased in ccRCC tissues compared with adjacent normal tissues. ccRCC patients with high UBE3C protein expression in tumors were associated with significantly worse postoperative survival. Knockdown of UBE3C expression in ACHN cells inhibited cell proliferation, migrations and invasiveness in vitro while overexpression of UBE3C in 786-O cells exerted the opposite effects. UBE3C up-regulated β-catenin protein levels and promoted β-catenin nuclear accumulation, leading to the activation of the Wnt/β-catenin signal pathway in RCC cells. Collectively, these observations suggest that UBE3C plays an important role in RCC development and progression, and UBE3C may be a novel target for prevention and treatment of ccRCC.

## Introduction

Renal cell carcinoma (RCC) arises primarily in the renal parenchyma, accounting for over 90% of kidney carcinomas, among which clear cell RCC (ccRCC) represents the most common histological subtype [1]. RCC has the highest mortality rate of the genitourinary cancers andthe incidence of RCC has risen steadily, accounting for approximately 200,000 new cases globally, with a mortality rate of more than 100,000 patients annually[2]. For treatment of localized RCC disease, nephrectomy is effective, whereas advanced RCC is still highly lethal with a 5-year survival rate of 53% [3]. Until now, insight into molecular mechanisms and pathways altered during the development of RCC remains limited. Hence, its elucidation will facilitate the identification of biomarkers for prognosis prediction and intervention.

Wnt family genes play important roles in human organogenesis and tumorigenesis; moreover, they are involved in renal development and initiation of several renal diseases including kidney malignancy [4–8]. the The β-catenin- dependent pathway or canonical pathway, is activated by Wnt-ligand binding to frizzled receptors (FZD) followed by cytosolic accumulation of β-catenin through prevention of glycogen syntheses kinase three β (GSK-3β) mediated phosphorylation of the β-catenin Ser/Thr domain. Only the canonical pathway activates the key regulator protein β-catenin phosphorylation-dependent degradation [9].As a result, increased reduced decreased cadherin-based cell adhesivity increased promoted epithelial-mesenchymal transition (EMT) and progressive cancer metastasis [10]. Meanwhile, Sstabilized β-catenin can translocate to the nucleus to initiate transcription of Wnt target-genes such as c-MYC and cyclin D1 by the way of interaction with T-cell factor (TCF)/lymphoid enhancer factor (LEF) transcription factors [9–11]. Activation of these genes results in increased cell proliferation and differentiation, reduced decreased cell- tocell adhesion, enhanced cell migration, and promotion of tumor formation [12–14]. Recent reports suggest that β-catenin overexpression in RCC is associated with high incidence rate and poor prognosis [15–18].

UBE3C is an E3 ligase with two characteristic domains: an IQ motif and a HECT domain. The mutations in the HECT domain of UBE3C often lead to pathophysiological states, including neurological disorders and human cancers [19–21]. There are few reports about the association between UBE3C and tumorigenesis.. Recently just one study reported that the high frequency of UBE3C mutations in HCC provides a preliminary connection between UBE3C and human cancer, UBE3C was over-expressed in HCC tissues and promoted HCC progression [22], extending its role in cancer development. The essential role of UBE3C in HCC development has prompted the interest in a potential connection between UBE3C and RCC. More importantly, whether UBE3C has a role in RCC growth, invasion and metastasis has never been investigated.

In this report, we demonstrate that UBE3C isa driving factor for ccRCC growth and metastasis. UBE3C expression was increased in ccRCC tissues compared with adjacent normal tissues. ccRCC patients with high UBE3C protein expression in tumors are associated with significantly worse postoperative survival. *UBE3C* promotes ccRCC cell proliferation, migration and invasiveness *in vitro*. UBE3C promotes proliferation, migration and invasiveness of ccRCC cells via activating the Wnt/β-catenin signal pathway.

## Materials and Methods

### Patients

Fresh tissue specimens from a series of 30 ccRCC patients who underwent resection in 2012 were obtained for real-time quantitative PCR and immunohistochemistry analysis. The tissue samples were snap frozen in liquid nitrogen immediately after resection and stored at -80°C until further analysis was performed.

In addition, archived formalin-fixed and paraffin-embedded tissue specimens derived from 267 consecutive cases of patients who had undergone radical nephrectomy or nephron-sparing surgery for unilateral, sporadic ccRCC from Jan. 2002 to Dec. 2005 at East Hospital of TongJi University were obtained, the matched peritumoral and tumor tissues were used to construct a tissue microarray for immunohistochemistry analysis. None of the patients had received chemotherapy or radiotherapy before surgery. After explaining the details of the study, potential benefits and risks associated with it, written informed consent was obtained from each included patient.

All of these patients agreed to participate in the study and gave written informed consent. Both this study and consent were approved by the the Ethics Committee of East Hospital and complied with the Declaration of Helsinki.

### Plasmid constructs

Flag-tagged full-length UBE3C cDNAs were cloned in pcDNA3 (Invitrogen) to create pFlag-UBE3C. Lentiviral vectors pLKO.1 TRC (Addgene plasmid 10879) and pWPI.1 (Addgene plasmid 12254) were used for producing recombinant lentiviruses. For RNA interference of UBE3C, DNA fragments encoding hairpin precursors for shUBE3C (5’-GCCAGACATTACTACTTCCTA-3’) was inserted into pLKO.1 TRC. A scrambled siRNA precursor (Scr) of similar GC-content to shUBE3C but no sequence identity with UBE3C was used as negative control. For overexpression of UBE3C, Flag-tagged UBE3C cDNA was cloned in pWPI.1.

### Cell lines, transfection and lentiviruses

Human RCC cell lines 786-O and ACHN were from Cell Bank of Shanghai Institutes of Biological Sciences, Chinese Academy of Sciences. Cells were maintained in Dulbecco’s modified Eagle medium (DMEM) (Invitrogen) supplemented with 100 U/ml penicillin G/streptomycin sulfate and 10% (v/v) fetal bovine serum (Invitrogen), and cultured at 37°C with 5% CO_2_.

The expression plasmids were transfected into cells using Lipofectamine 2000 (Invitrogen) according to the manufacturer’s instructions.Helper plasmids pSPAX2 (Addgene plasmid 12260) and pMD2.G (Addgene plasmid 12259) were co-transfected with pLKO.1- or pWPI.1-based plasmids into HEK293T cells to package recombinant lentiviruses. Supernatants from co-transfections were used for infection of cultured cells.

### RNA isolation and quantitative real-time PCR (qrtPCR)

Total RNA was extracted using TRIzol Reagent (Invitrogen), and reverse transcription was performed using PrimeScript RT reagent Kit (TaKaRa) according the manufacturer’s instructions. The corresponding primer sequences were as follows: UBE3C sence primer: GTGCCCGGCTGCTTCCGCGGC; reverse primer: CCTCCTTCCTGCTCGCGCCGCC. β-actin sence primer: TCCCTGGAGAAGAGCTACG; reverse primer: GTAGTTTCGTGGATGCCACA. For qrtPCR analysis, aliquots of cDNA were amplified using SYBR Premix Ex Taq (TaKaRa). PCR reactions were done in triplicates with following conditions: 95°C/30 s, 40 cycles of 95°C/5 s, 60°C/15 s and 72°C/10 s on MXP3000 cycler (Stratagene) and repeated at least three times. Relative mRNA levels were calculated using the -ΔΔCt method using *β-actin* as control and expressed as 2^(-ΔΔCt).

### Cell growth and proliferation

Cell proliferation was measured using a CCK-8 kit (Dojindo, kumamoto, Japan). RCC cell lines infected with Lentiviruses were seeded into 96-well plates in 100 µl of medium containing 10% FBS and incubated at 37°C in 5% CO_2_. After 24, 48, 72 and 96 h, the medium was replaced with 90 µl of fresh medium and 10 µl CCK-8 solution was added to each well. Cells were then incubated for 2 h at 37°C in 5% CO_2_, after which absorbance at 450 nm was measured using a microplate reader (Molecular Devices, CA, USA). Each experiment was performed in triplicate and repeated in quadruplicate for each condition.

### Colony-formation assay

786-O and ACHN cells infected with lentiviruses were seeded separately in six-well plates at a density of 1×10^2^ cells/well. After incubation at 37°C for ~10–14 d, cells were washed twice with PBS, stained with Giemsa solution (AppliChem, Darmstadt, German), and allowed to air dry at room temperature. The number of colonies was microscopically counted, Each experiment was performed in triplicate.

### Transwell cell migration and invasion assays

For transwell migration assays, cells were plated in the top chamber with the non-coated membrane (24 well insert; 8μm, Corning Costar Corp). For invasion assays, matrigel (BD biosciences) was allowed to polymerize at the base of the top chamber of a 24 well transwell plate (for 45 minutes at 37°C. 786-O and ACHN cells or its derivative cells (1×10^5^ cells/well) were starved in serum- and growth factor-free medium for 24 hours and added to the top chambers. The bottom chambers were filled with serum-containing medium. Cultures were maintained for 48 hours, followed by removal of non-invading cells from the upper surface of the membrane with cotton swabs. Cells that invaded were fixed in 4% paraformaldehyde and stained with 0.1% crystal violet for 15 minutes at room temperature. Excess dye was washed off.

### Luciferase reporter assay

Exponentially growing cells were seeded 24 h prior transfection at 1.0×10^5^ cells/well (≈80% confluence) into 12-well tissue culture plates. Transient co-transfection with luciferase construct (pLEF-luc, pC/EBP-luc), pSV-β-galactosidase vector, expression vector (i.e., UBE3C, shUBE3C) or their corresponding empty vectors controls was carried out using the Lipofectamine 2000 Reagent (Invitrogen, Carlsbad, CA, USA). The DNA/Superfect ratio was 1:7.5 (wt/wt). After incubation for 48 h, cells were assayed using the Luciferase reporter assay system kit (Promega, Fitchburg, WI, USA), according to the manufacturer’s instructions. The promoter activity was calculated from the ratio of firefly luciferase to β-galactosidase levels and expressed as arbitrary units. All experiments were performed in triplicate and data were pooled from three independent experiments.

### Immunohistochemistry

Sections of 5-*μ*m thickness were deparaffinised in xylene and rehydrated through graded alcohol series. For antigen retrieval, the sections were heated in EDTA buffer (pH9.0) for 20 min at 100°C. The tissue sections were then treated with 0.3% H_2_O_2_ for 5 min to block the endogenous peroxidase activity and subsequently rinsed with phosphate-buffered saline (PBS) for 3 times, 2 min each time. Rabbit anti-UBE3C antibody (ABclonal) was used to detect UBE3C expression. The antibody was diluted 1:100 in TNB blocking solution and incubated with the samples overnight at +4°C. After rinsing with PBS, the tissue sections were then incubated for 30 min with mouse anti-rabbit secondary antibody. The slides were washed with PBS again, and 3,3’-diaminobenzidine (DAB) was used as a chromogen to visualize the reaction. Finally, the sections were counterstained by hematoxylin, dehydrated and mounted in Diatex. Negative control immunostaining was performed by omission of the primary antibody. Hepatocellular carcinoma (HCC) specimen was examined as positive control.

### Evaluation of immunohistochemical staining

UBE3C expression was evaluated by two investigators blinded to the clinicopathological data of the patients. Sections were considered to be positive when tumor cells showed cytoplasmic or nuclear UBE3C expression. Every tumor was given a score by multiplying the percent of stained cells (0, 0%; 1, less than 25%; 2, 25–50%; 3, more than 50%) with the staining intensity (0, no staining; 1, weak staining; 2, moderate staining; 3, strong staining). We then designated total score 0–3 as low expression, whereas total score 4–9 as high expression.

### Statistical Analysis

Statistical analysis was performed with SPSS 13.0 for Windows (SPSS Inc). The Pearson *χ* ^2^ test or Fisher’s exact test was used to compare qualitative variables, and the Student *t* test for quantitative variables. All statistical tests were two-sided, and *p* value < 0.05 was considered statistically significant.

## Results

### UBE3C high expression in ccRCC

**Figure 1:**
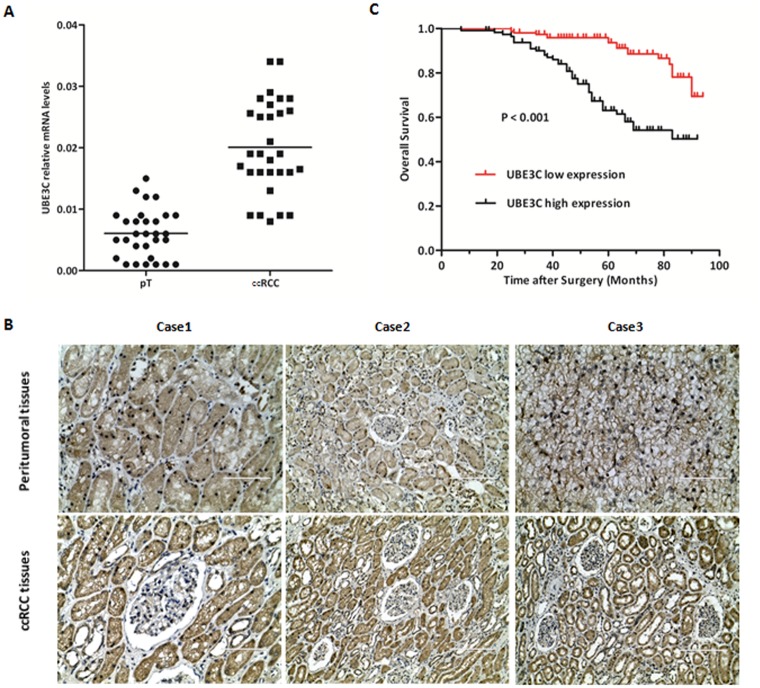
The prognostic significance of UBE3C expression in ccRCC. (A) Real-time quantitative RT-PCR analysis of UBE3C expression in ccRCC and corresponding peritumoral renal tissues. The relative expression of UBE3C mRNA in ccRCC tissue samples was higher than that in the paired peritumoral renal tissues. (B) Representative immunostaining of UBE3C expression in ccRCC was higher than corresponding peritumoral renal tissues. (C) Kaplan-Meier curves of OS according to UBE3C expression in ccRCC. The OS was significantly decreased in the high UBE3C expression group compared to the low UBE3C expression group (Log-Rank test, *p*<0.001).

Expression of UBE3C mRNA was first assessed in 30 ccRCC specimens and matched peritumoral renal tissues, 76.7% (23/30) specimens presented over-expressed UBE3C. UBE3C expression was significantly increased in ccRCC tissues than in matched peritumoral renal tissues (Fig. 1A) (P = 0.003). Additionally, a UBE3C expression study revealed that UBE3C protein was expressed in the cytoplasm, and UBE3C protein levels were significantly higher in ccRCC tissues than in paired peritumoral tissues (Fig. 1B).

### The prognostic significance of UBE3C expression in ccRCC

We analysized the UBE3C protein expression in tumor specimens derived from 267 postoperative ccRCC patients whose 5-year follow-up data were available. The specimens could be grouped under UBE3C high expression (189/267, 70.8%) or UBE3C low expression according to immunohistochemistry staining scores. The correlation between UBE3C high expression and TNM stage was also evaluated. It was found that in T1a group, the percent of over-expressed UBE3C was 38.6% (17/44); in T1b group, the percent was 67.0% (67/100); in T2 group, the percent was 80.4% (45/56); in T3–T4 group, the percent was 91.0% (61/67). The UBE3C over-expression in ccRCC tumor tissues showed a positive correlation with TNM stage (r = 0.731, P<0.01). Next, we evaluated the associations between the UBE3C expression and overall survival (OS) by Kaplan-Meier survival analysis with log-rank statistic for determining significant difference. The OS was significantly decreased in the high UBE3C expression group compared to the low UBE3C expression group (Fig. 1C and S1 File).

### UBE3C promotes RCC cell proliferation in vitro

**Figure 2:**
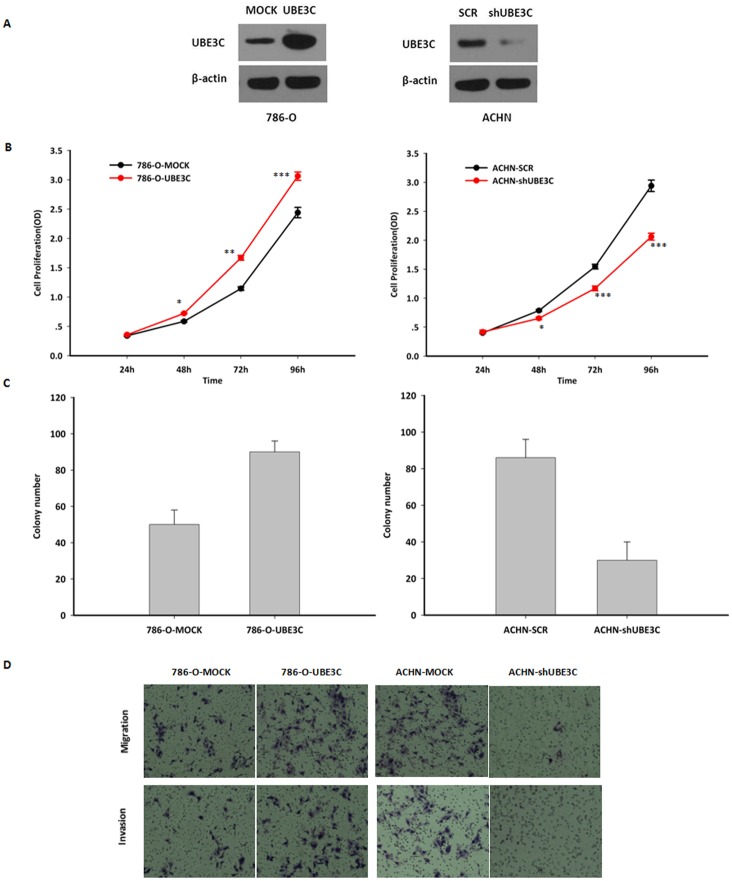
UBE3C improves RCC cell proliferation, cell migration and invasiveness in vitro. (A) UBE3C expression of RCC cells infected with lentivirus. Left: Overexpression of UBE3C in 786-O cells. Right: Knockdown of UBE3C expression in ACHN cells. (B) The CCK-8 assay revealed that UBE3C overexpression significantly improved the cell growth rate (Left). Knockdown of UBE3C expression in ACHN cells significantly reduced the cell growth rate. Means±SD from three independent experiments were presented. (C). Left: Overexpression of UBE3C expression promoted cell colony-forming capacity in 786-O cells. Right: knockdown of UBE3C expression inhibited cell colony-forming capacity in ACHN cells. Means±SD from three independent experiments were presented. (D) Left: Overexpression of UBE3C expression promoted cell migration and invasion in 786-O cells. Right: Knockdown of UBE3C expression significantly inhibited cell migration and invasion in Matrigel transwell assays. The permeable cells were stained with Giemsa. Significant differences were determined using Student’s t test, *P<0.05, **P<0.01, ***P<0.001.

Lentivirus-mediated overexpression of *UBE3C* expression in the 786-O cells and lentivirus-mediated knockdown of *UBE3C* expression in the ACHN cells were performed to assess the functional involvement of UBE3C in RCC cell proliferation in vitro. Re-expression of UBE3C in stably overexpressed 786-O cells and knockdown ACHN cells were confirmed by western blot analysis (Fig. 2A). Overexpression of UBE3C promoted the growth of the 786-O cells over a course of 96 hours and increased the number of 786-O cells colonies, as determined by colony formation assay (Fig. 2B–2C). In contrast, knockdown of *UBE3C* expression in ACHN cells reduced cell growth (Fig. 2B) and colony formation (Fig. 2C). Taken together, these data suggest that UBE3C promotes the proliferation of RCC cells.

### UBE3C promotes RCC cell migration and invasiveness in vitro

In order to assess the functional involvement of UBE3C in RCC cell invasion *in vitro*, we used the above stably cells to tested cell migration and invasiveness using wound-healing and transwell invasion assays. The control cells were infected with the lentivirus expressing scrambled siRNA precursor. Compared with the control cells, ACHN-*shUBE3C* cells displayed sharp declines in cell migration and invasiveness (Fig. 2D). In contrast, exogenous expression of *UBE3C* in 786-O cells (Fig. 2A) enhanced cell migration and invasiveness markedly (Fig. 2D). These results indicate that UBE3C promotes RCC cell migration and invasiveness *in vitro*.

### UBE3C up-regulates β-catenin protein levels and promotes β-catenin nuclear accumulation

**Figure 3:**
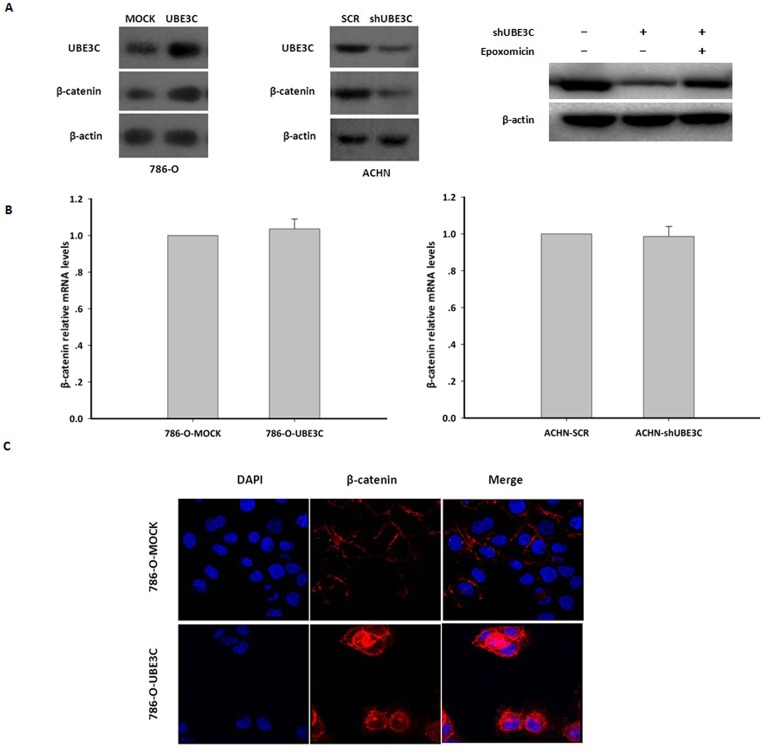
UBE3C up-regulates β-catenin expression and promotes the nuclear accumulation of β-catenin. (A) Overexpression of *UBE3C* in 786-O cells increased β-catenin expression (left). But, knockdown of *UBE3C* expression in ACHN cells reduced β-catenin expression (middle). β-catenin degradation induced by UBE3C knockdown was substantially attenuated when the cells were treated with epoxomicin (right). (B) Overexpression of UBE3C in 786-O cell did not change β-catenin mRNA expression. Knockdown of UBE3C expression also did not induce β-catenin mRNA levels. β-catenin mRNA was measured using qrtPCR. Means±SD from three independent experiments were presented as relative ratio to the control whose value was taken as 1.0. (C) Immunofluorescence assay revealed that exogenous expression of UBE3C indeed promotes β-catenin translocation to the nucleus in 786-O cells.

The canonical Wnt/β-catenin pathway is one of most frequently involved in the initiation and progression of ccRCC [4–6]. We postulated that UBE3C activates Wnt/β-catenin signal pathway in order to promote tumor growth and metastasis in RCC cells. Because β-catenin is the central player of the Wnt/β-catenin signaling pathway, whether UBE3C can regulate β-catenin protein levels in RCC cells was firstly investigated. Overexpression of UBE3C up-regulated β-catenin protein levels in 786-O cells (Fig. 3A); in contrast, knockdown of UBE3C down-regulated β-catenin protein levels in ACHN cells (Fig. 3A). However, in *UBE3C* overexpression 786-O *cells and UBE3C*-knockdown ACHN cells could not change *β-catenin* mRNA levels (Fig. 3B and S2 File). To test whether UBE3C influence β catenin protein stability, we used epoxomicin to block proteasome-mediated protein degradation and found that βncatenin degradation induced by UBE3C knockdown was substantially attenuated after epoxomicin-treatment (Fig. 3A). Immunocytofluorescence further showed that exogenous expression of UBE3C promoted β-catenin translocation to the nucleus in 786-O cells (Fig. 3C). Taken together, these data suggest that UBE3C can up-regulate β-catenin protein level and promote β-catenin nuclear accumulation in RCC cells.

### UBE3C activates Wnt/β-catenin pathway

**Figure 4:**
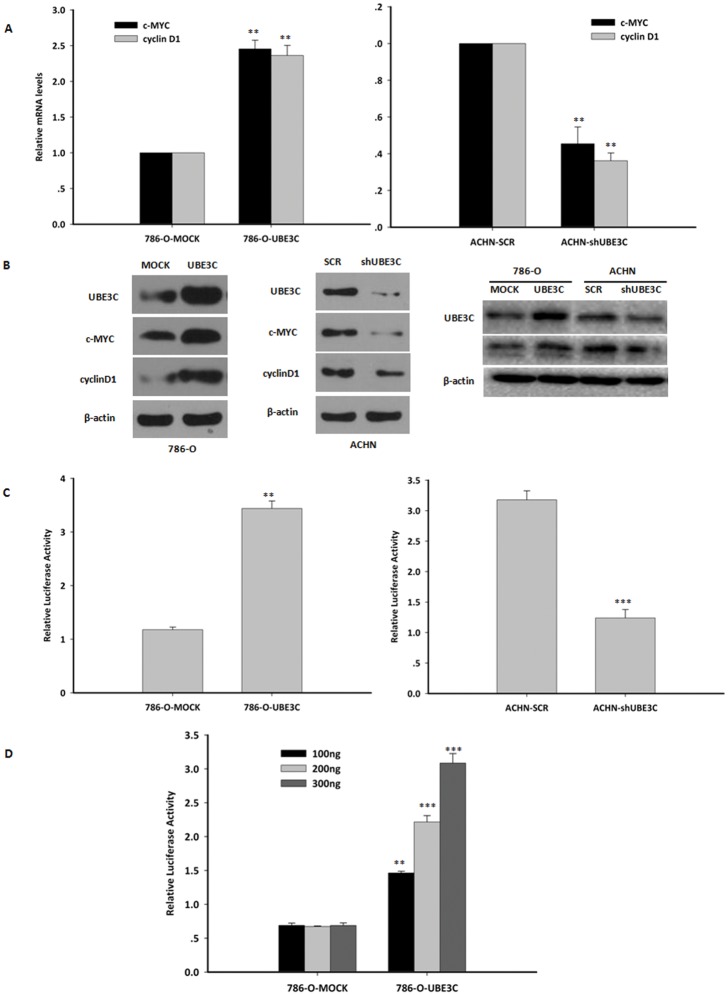
UBE3C activates Wnt/β-catenin pathway. (A) Left, *UBE3C* overexpression in 786-O cells up-regulated c-MYC and cyclin D1 mRNA levels. Right, *UBE3C* knockdown in ACHN cells down-regulated c-MYC and cyclin D1 mRNA levels. Means±SD from three independent experiments were presented as relative ratio to the control whose value was taken as 1.0. (B) Left, overexpression of UBE3C increased c-MYC and cyclin D1 protein levels in 786-O cells. Middle, knockdown of UBE3C decreased c-MYC and cyclin D1 protein levels in ACHN cells. Right, overexpression of UBE3C increased p-GSK3β level in 786-O cells, meanwhile knockdown of UBE3C decreased p-GSK3β level in ACHN cells. (C) Left, overexpression of UBE3C up-regulated the LEF reporter activity in 786-O cells. Right, *UBE3C* knockdown down-regulated the LEF reporter activity in ACHN cells. Means±SD of normalized luciferase activity from three independent experiments were presented. (D) UBE3C activated the LEF reporter in dose-dependent manners. 786-O cells were co-transfected with the expression constructs of *UBE3C* and the LEF reporter. Significant differences were determined using Student’s t test, *P<0.05, **P<0.01, ***P<0.001.

The UBE3C-mediated up-regulation of β-catenin protein levels and nuclear accumulation in RCC cells suggests that UBE3C might activate the Wnt/β-catenin pathway in RCC cells. To tackle this issue, we began by investigating whether exogenous expression of UBE3C caused any change in Wnt/β-catenin target genes, c-MYC and cyclin D1. Overexpression of *UBE3C* expression significantly increased c-MYC and cyclin D1 mRNA levels in 786-O cells (Fig. 4A and S2 File). On the contrary, knockdown of UBE3C reduced c-MYC and cyclin D1 mRNA levels in ACHN cells (Fig. 4A). This effect might increase c-MYC and cyclin D1 protein levels.A, western blot analysis showed that overexpression of UBE3C up-regulated c-MYC and cyclin D1 protein levels (Fig. 4B), but knockdown of UBE3C down-regulated c-MYC and cyclin D1 protein levels (Fig. 4B). Next, we wondered whether exogenous expression of UBE3C can activate LEF luciferase reporter. To determine whether UBE3C activates LEF luciferase reporter, 786-O cells were co-transfected with the expression constructs for *UBE3C* and *LEF* reporter system. In 786-O cells, UBE3C was able to activate *LEF* promoter as revealed by luciferase reporter assays (Fig. 4C and S3 File). Moreover, this activation was in a dose-dependent manner in 786-O cells (Fig. 4D). In ACHN cells, knockdown of UBE3C expression reduced *LEF* promoter activity (Fig. 4C and S3 File). In addition, to investigate whether UBE3C directly activate the Wnt/β-catenin pathway, we evaluated phosphorylation (ser9) level of GSK3β when UBE3C overexpression or knockdown, for a hallmark of the pathway activation is the accumulation of β-catenin protein in the cytoplasm, in which inhibition of GSK3β activity by phosphorylation can result. It was found that phosphor- GSK-3β (Ser9) increased in UBE3C over-expressed 786-O cells, while decreased in UBE3C knockdown ACHN cells (Fig. 4A). Taken together, these data showed that UBE3C could activate the Wnt/β-catenin pathway in RCC cells.

## Discussion

RCC is the most common primary malignancy of the kidney and one of the most lethal genitourinary malignancies [22]. ccRCC has an extremely poor prognosis because of a high potential for vascular invasion, metastasis and recurrence [23–25]. Despite this the mechanism of ccRCC growth and metastasis is not well understood, evidence has been presented to indicate that Wnt/β-catenin signaling pathway is critical for ccRCC growth and metastasis [12, 17]. In this study, we demonstrate that UBE3C activates Wnt/β-catenin pathway and consequently induces proliferation and metastasis of the ccRCC cells. Consistent with these discoveries, postoperative ccRCC patients who had relatively high UBE3C levels in tumors are associated with significantly worse survival. Collectively, we demonstrate UBE3C as a driving factor for ccRCC growth and metastasis.

UBE3C is an E3 ligase which contains two protein domains: an IQ motif and a HECT domain. The IQ motif mediates substrate targeting [21], but the HECT domain binds to ubiquitin-conjugating enzymes (E2) and mediates ubiquitin to the target substrate [26]. Therefore, the HECT domain of the UBE3C provides the E3 ligase catalytic activity. E3 ligase performs pivotal roles in the cell cycle, DNA repair, cellular homeostasis, and biological signaling via selectively binding to their protein substrates. The dysfunction of the HECT E3 ligase contributes to pathological disorders, including various kinds of human carcinomas [27]. E6AP, the first identified member of the HECT E3 family [28], targets the tumor suppressor protein p53 via ubiquitin-mediated proteolysis, which improves p53 protein degradation and leads to the development of the human cervical cancer [29]. Recently, many tumor suppressor molecules have been reported as substrates of HECT E3 ligase and genetic aberrations in and dysexpression of the HECT E3 ligase is frequently observed in various kinds of human cancers [30–32]. As to our known, there is no study reporting correlation of UBE3C and ccRCC. In this study, we confirmed that UBE3C expression was markedly increased in ccRCC tissues compared with adjacent normal tissues. Additionally, we also found that ccRCC patients with high UBE3C protein expression in tumors are associated with significantly worse postoperative survival. Nevertheless, these results indicate that UBE3C protein expression may be a promising prognostic biomarker for ccRCC.

The Wnt/β-catenin signaling pathway has been reported to be associated with various kinds of human carcinomas including ccRCC [4–6]. β-catenin is considered as a positive regulator of this pathway, mediated by promoting transcription of target genes. Some target genes are involved in oncogenesis, such as c-MYC and cyclin D1 regulating cancer proliferation, MMP-7 regulating cancer invasion [12–14]. To the best of our knowledge, there is no study that infers the relationship between UBE3C and Wnt/β-catenin pathway in ccRCC. In this study, we demonstrate for the first time that overexpression of UBE3C promotes cell growth, migrations and invasiveness of the RCC cell line 786-O in vitro; in contrast,. However, knockdown of UBE3C expression inhibits cell growth, migration and invasiveness of the RCC cell line ACHN in vitro. Meanwhile, Ooverexpression of *UBE3C* expression significantly increased c-MYC and cyclin D1 mRNA and protein levels in 786-O cells. On the contrary, knockdown of UBE3C reduced c-MYC and cyclin D1 mRNA and protein levels in ACHN cells. Exogenous expression of UBE3C can activate LEF luciferase reporter in a dose-dependent manner in 786-O cells. In addition, UBE3C could stabilize the cytoplasmic β-catenin and promote its translocation into the nucleus through enhancing GSK-3β phosphorylation and inhibiting its activity. Altogether, these results demonstrate that UBE3C may be associated with cell proliferation, migration and invasiveness of ccRCC via activating Wnt/β-catenin signaling pathway.

Given the extremely poor prognosis of ccRCC, biomarkers for prediction and intervention are urgently needed. So far, there has no specific biomarker used in clinical diagnosis and prediction of prognosis. To our knowledge, the present study examined for the first time the possibility of using UBE3C as a clinically potential indicator for disease progression, as well as a prognostic marker for patient survival in ccRCC. Moreover, our findings revealed that UBE3C expression was increased in ccRCC tissues compared with adjacent normal tissues. ccRCC patients with high UBE3C protein expression in tumors are associated with significantly worse postoperative survival. In agreement with clinical findings, in vitro cell experiments confirmed that UBE3C promotes RCC cell proliferation, migration and invasiveness via activating the Wnt/β-catenin signal pathway. Consequently, UBE3C plays an important role in RCC development and progression, and UBE3C may be a novel target for prevention and treatment of ccRCC.

## Supporting Information

S1 File The relationship between UBE3C expression and overall survival time in ccRCC patients. The evaluation of UBE3C expression was carried out through immunohistochemical staining. The high expression of UBE3C was labeled as 1 and the low expression as 0. Then the relationship between UBE3C expression and overall survival time was analyzed through Kaplan-Meier curves.
(XLSX)

S2 File The Ct values of various treated cells by real-time PCR analysis. After knockdown or overexpression of *UBE3C* in ACHN or 786-O cells, the *c-myc*, *cyclin D1* and *CTNNB1* mRNA were analyzed by real-time PCR.
(XLSX)

S3 File Activation of the LEF reporter by UBE3C in dose-dependent manners. In LEF reporter luciferase assay, 786-O cells were respectively treated by 100ng, 200ng and 300ng UBE3C plasmid. The promoter activity was calculated from the ratio of firefly luciferase to β-galactosidase levels and expressed as arbitrary units.
(XLSX)
